# Mechanisms of Action and Efficacy of Statins against Influenza

**DOI:** 10.1155/2014/872370

**Published:** 2014-11-11

**Authors:** Parvaneh Mehrbod, Abdul Rahman Omar, Mohd Hair-Bejo, Amin Haghani, Aini Ideris

**Affiliations:** ^1^Institute of Bioscience, Universiti Putra Malaysia, 43400 Serdang, Selangor, Malaysia; ^2^Faculty of Veterinary Medicine, Universiti Putra Malaysia, 43400 Serdang, Selangor, Malaysia

## Abstract

The influenza virus (IV) is known to be a resistant virus with frequent mutations, causing severe respiratory diseases in the upper respiratory system. Public health concerns about clinical efficacy of all conventional drugs are ambiguous; therefore, finding additional therapeutic agents is critical to prevent and control influenza outbreaks. Influenza is associated with the induction of proinflammatory cytokines. Scientists have reported that anti-inflammatory drugs, with pleiotropic effects, reduce the burden of severe influenza diseases. Therefore, statins, which are cardioprotective drugs with anti-inflammatory and immunomodulatory effects, may help patients suffering from influenza virus (IV). This review delineates the potential use of statins as an alternative therapy in treating influenza related illness.

## 1. Statins

Statins are hydroxyl methylglutaryl-coenzyme A (HMG-CoA) reductase enzyme inhibitors. This enzyme is responsible for the rate determining in early step of cholesterol biosynthesis pathway [1], which catalyses the reduction of HMG-CoA to mevalonate during the synthesis of cholesterol [2]. Statins are pleiotropic cardioprotective drugs, increasingly recognized as mediators of direct cellular effects beyond their lipid lowering capacity [3]. Statins can block downstream molecules which are key factors in virus infectivity [4]. They reduce or block the synthesis of downstream isoprenoid intermediates, such as farnesyl pyrophosphate (FPP) and geranylgeranyl pyrophosphate (GGPP) [5]. These isoprenoid intermediates are lipid attachments for the posttranslational modification of proteins that interact with membranes. Statins manage their GTP- and GDP-bound state by controlling their isoprenylation and inhibiting the movement of their inactive form from cytosol to the membrane [6].

Therefore, statins that decrease the synthesis of isoprenoid intermediates, inhibit their membrane translocation and activity [6, 7]. Pleiotropic effects of statins are thought to be mediated by reduction in the isoprenylation of many proteins. This can be illustrated by the Rho GTPase family: RhoA, Rac, and Cdc42 that serves specific functions in terms of cell shape, motility, secretion, proliferation, and regulation of gene expression [6]; Ras family also requires an attachment of a farnesyl group for their actions and membrane associations to play a role in cellular differentiation and proliferation [8]; Rab family, which are necessary for vesicle transportation inside the cell [8]; and Rap family, which are known to play a role in cell replication, oxygen radicals generation and platelet activation [7]. The diagram shows the pathways of statin effects on cellular and molecular structures that may interfere with influenza pathogenesis.

A considerable amount of literature has discussed pleiotropic effects of statins on leukocyte-endothelial interaction, intra- and intercellular signaling, inflammatory gene transcription, hemoxygenase expression, and expression of MHC class II antigens [9, 10]. In addition, anti-inflammatory and immunomodulatory properties have been shown by many other studies [9, 11–14].

Thus, statin drugs have captivated attention to have clinical significance in the preventative treatment averse to cellular damage caused by infectious agents [15, 16]. Furthermore, statins can reduce sepsis and infections associated with complex inflammatory reactions, which is by virtue of the diverse and extrabeneficial anti-inflammatory effects that are independent of their lipid-lowering ability [17].

## 2. Influenza Virus (IV)

Influenza A virus is the causative agent for respiratory infections. Since 2009, a new reassorted A/H1N1 virus had circulated worldwide among humans, causing morbidity and mortality, and was referred to as a pandemic H1N1 (pH1N1) [18, 19]. The clinical manifestations and the severity of illnesses following influenza infections are the result of immune dysregulations. This can be exemplified by release of proinflammatory cytokines that cause severe complications like hypercytokinemia [20, 21]. This condition can be terminal in some strains like H5N1 [22, 23] and H1N1 [24].

The currently-approved classes of antiviral drugs for the treatment of influenza viral infections include neuraminidase inhibitors (zanamivir and oseltamivir) and M2 channel blockers (amantadine and rimantadine) [35]. Resistance to both classes is a steady dilemma. A widespread resistance to the only orally bioavailable neuraminidase inhibitor, oseltamivir, was encountered in the 2008-2009 [36] and 2009-2010 influenza seasons [37]. Although amantadine was successfully used for more than three decades, widespread resistance against this compound has made the Center for Disease Control and Prevention (CDC) in the US to advise against continuing usage of this drug [38]. Due to the fact that the next occurrence of influenza epidemic or pandemic is unpredictable, it is required to have an effective treatment strategy.

However, no new classes of medications have convincingly demonstrated the characteristics to ameliorate the clinical outcomes of influenza infection. Therefore, there is an imperative need for the development of anti-influenza drugs with broad reactivity against all strains and subtypes. Hence, it is necessary to contemplate the option of moving into multiple drug therapy. Moreover, finding effective alternative therapy to reduce the complications of influenza is required [16, 39]. In this respect, other effective and safe drugs, which bypass the possibility of developing resistance, are eminently demanded to limit the severity of clinical health problems associated with influenza A virus infections. An ideal alternative drug should be low cost generic medications, easily accessible in pandemics events. This type of drug should aim at the host's immune response to alleviate the effects of influenza and also exhibit ameliorated efficacy and safety by targeting required cellular proteins for influenza virus replication [39–41]. One of the advantages of this approach is avoiding development of drug resistance. Such drugs target common pathways used by host and influenza virus [42, 43]. Since influenza A viruses widely use the host cell machinery to support the replication and transportation of their own viral components [20], targeting the host's response for exclusively attacking the virus could be advantageous. However, this strategy requires a better enlightenment of the intracellular pathways by which the influenza virus replicates [42].

**Figure 1:**
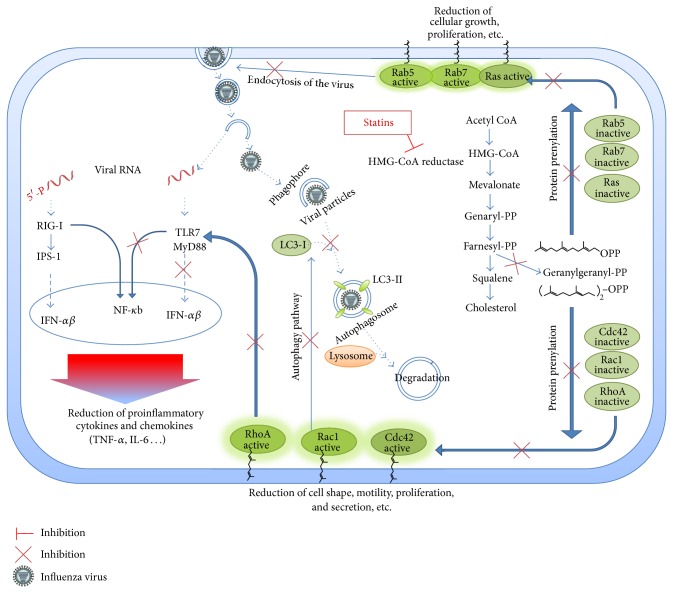
Possible mechanisms of statin action against influenza A virus pathogenesis in cellular and molecular structures.

Therefore, anti-inflammatory and immunomodulatory agents can be effective alternative therapeutics to vaccines and conventional antiviral drugs [16]. For the first time, the idea of statin usage to reduce pandemic mortality was suggested by Fedson [16] and, recently, these immunomodulatory agents have attracted much attention [44]. Statins can block downstream molecules that are key factors in virus infectivity [4]. These compounds are promising alternatives for influenza treatments by limiting inflammation, preventing cellular destruction [45]. Influenza pathogenesis and immune response of the infected cells with more focus on the statins mechanisms of action are shown in Figure 1.

## 3. In Vitro Study

Statins represent one of the most widely prescribed drugs in the world; therefore, identifying their capacity to reduce influenza virus mortality and morbidity during a pandemic is a matter of public health importance. However, in vitro studies regarding the effects of statins against influenza virus are limited.

Haidari and collaborators tested statin's inhibitory effects on the Rho/Rho kinase pathway, which led to the inhibition of influenza virus proliferation, with the use of atorvastatin and rosuvastatin against H3N2 and H1N1 strains in Madin-Darby canine kidney (MDCK) cells [40]. They showed the involvement of Rho/Rho kinase pathway in virus proliferation, through which statins exhibit anti-influenza effects by downregulating the Rho/Rho kinase pathway [40]. The anti-inflammatory effects of statins against H1N1 infection in Crandell feline kidney (CrFK) cells have also been recently investigated [12]. It was found that atorvastatin, simvastatin and pravastatin were equally effective in reducing the expression levels of proinflammatory cytokines such as TNF-*α* and IL-6. In addition, atorvastatin-, simvastatin-, and pravastatin-treated H1N1 infected cells showed significant decrement in the expression of proinflammatory cytokine proteins, compared to the virus infected cells alone [12]. Therefore, statins might be considered as a competent supplementary to conventional therapeutics in controlling the cytokines' overproduction.

The study by Lee et al. [14] demonstrated the attenuation of viral dsRNA-induced AKT phosphorylation, STAT3 activation, and the subsequent production of RANTES using simvastatin treatment in primary normal human bronchial epithelial cells (NHBE) and a human type II pneumocyte cell line A549.

Recently, it was shown that atorvastatin is as effective as amantadine and oseltamivir in reducing viral titer and augmenting cell viability following infection with the H1N1 strain in MDCK cells [46]. Another study demonstrated that H1N1 infection activates RhoA protein prenylation and induces actin cytoskeleton remodeling. Hence, simvastatin may reduce the replication of H1N1 by the possible blocking of RhoA membrane localization and inducing actin filaments condensation. Consequently, simvastatin can affect the upregulated RhoA pathway induced by H1N1 [47]. Simvastatin also showed analogous effects on the expression of Rab proteins during endocytosis. In that study, LC3-II protein localization, an important marker involved in the autophagy process, was affected by simvastatin which contribute to retardation in the maturation process of autophagosomes [47].

All in all, in vitro studies showed the possible promising functions of atorvastatin, simvastatin, and pravastatin as the representatives of the statin family, on several cellular pathways to block the virus-host interaction system, which can act as an effective compound opposed to H1N1 infection.

## 4. Animal Study

Selected animal studies have further recommended a protective role for statin drugs against acute lung injury; however, results have been confounding. Gower and Graham in 2001 tested lovastatin against the respiratory syncytial virus and proposed lovastatin as an antiviral agent that controls the RhoA membrane localization and affects virus replication [48]. Jacobson et al. [49] confirmed significant protection by simvastatin on LPS-induced lung vascular inflammation and implicated a potential role for statins in the management of acute lung injury; however, Ferraro et al. [50] adverted to nonsignificant effect of simvastatin on inflammatory biomarkers in lung injuries. Nevertheless, Haidari et al. [40], who examined atorvastatin on C57BL/6 mice infected with H3N2 and H1N1, showed reduced lung virus titers and mortality rates. In addition, several subsequent studies have shown partial benefits of statin in the recovery from influenza virus infection. However, these studies have not generally engaged contemporary, wild-type viruses and have not widely examined statin drugs along with commercially available antiviral treatments [51–53].

The domination of cytokines' overexpression during influenza virus infection is a debatable topic. Two separate studies on experimental infections using knockout mice showed poor efficacy of treating influenza infections with immunomodulatory agents [54, 55]. They deduced that overproduction of cytokines might not be the cause of death during the H5N1 disease. Early inhibition of viral replication might be more promising in promoting survival of influenza virus infection than inhibition of the cytokine response. However, these two studies came from experiments conducted on small numbers of knockout mice, which does not provide sufficient basis for a conclusion.

Liu et al. [56] conducted a study in a murine model to evaluate the effectiveness and safety of a novel statin/caffeine mixture opposed to H5N1, H3N2, and H1N1 influenza virus infections. Caffeine is a constituent of the methylxanthine family [57], which has shown potential therapeutic effectiveness [58]. Caffeine modulates both innate and adaptive immune responses and prolongs the analgesic activities of painkillers, along with the effects of many other drugs [59]. In this study conducted by Liu et al. administration of 50 *μ*g statin/200 *μ*g caffeine mixture was as effective as oseltamivir and ribavirin in inhibiting virus replication and improving lung damage.

Recently, two studies that have evaluated the efficacy of statin treatment in influenza virus-infected mice showed that treatments had no effect on survival. Their experiments flaws could be related to choosing improper lethal dose of the viruses in the short treatment duration [34, 52].

In a study, Belser et al. [60] inoculated the BALB/c mice with H5N1 or pH1N1 after an oral administration of simvastatin and oseltamivir to determine the susceptibility of different influenza viruses to statin treatment. The blood has been analyzed for circulating lymphocytes, virus titration, and proinflammatory cytokines and chemokines. No prominent antiviral activity of simvastatin alone or in combination therapy has been found in comparison with solely oseltamivir therapy in mice. However, reduced hypercytokinemia following H5N1 but not pH1N1 infections in mice, after simvastatin administration, reinforces the notion of dependence of this treatment on virus strain. This study supports further research on using statin to ameliorate severe influenza diseases. Glück and colleagues also showed failure of simvastatin to protect mice against influenza virus infection [61]. They administered simvastatin in two different ways orally and/or intraperitoneally once daily, starting 3 days before virus infection until 14 days post infection. However, in any dosage or route of administration simvastatin did not enhance the survival rate.

Meanwhile, Lee et al. [14] developed a dsRNA-induced viral pneumonia mouse model and delivered statins by intranasal route, which allowed direct contact with the respiratory epithelium. They demonstrated that statins decreased STAT3 and RANTES expression in airway epithelia as compared to controls. Statins also attenuated the proinflammatory cytokine response and decreased neutrophil influx. Their findings were in accord with earlier published studies [62]. As shown in animal studies, statin treatments may be of particular benefit in severe cases of influenza virus infections by reducing influenza virus-induced pulmonary inflammation. However, further studies are required to elucidate the proper usage of this treatment approach as opposed to different virus strains.

## 5. Human Study

Despite contradictory reports, several retrospective observational studies have identified a connection between statin use and reduction of influenza virus morbidity in humans; however, considerable variability in timing and duration of drug administration among participants may restrict the results of these studies [25, 26, 32, 63].

Two epidemiological studies evaluated the statin/influenza hypothesis. One was conducted in the Netherlands, using a general administrative database. The other used the United Kingdom's General Research Database. They found that patients who were under statin treatments for the prevention of cardiovascular diseases were protected against influenza-associated symptoms, with reductions in influenza-related pneumonia, acute myocardial infarction, and stroke [64]. Later, in another major study, Frost et al. [25] published a study using a health maintenance organization, encountering data from several moderate-sized health maintenance organizations in New Mexico. They found 40% lower mortality rate of pneumonia and influenza in the recipients of moderate doses of statins (≥4 mg/day) compared to nonrecipients. Few years later, another study in Mexico showed higher survival rate in cases treated with statin (pravastatin 40 mg/day) which have been reported in a small number of influenza patients admitted to an intensive care unit [28]. However, simultaneously a cohort study by Viasus et al. [29] on pneumonia patients in Spain did not show any improvement by anti-inflammatory therapy. Up to this point, two randomized controlled trial (RCT) found beneficial effects of oral administration of statin in reducing the frequency of viral pneumonia [30, 65]. However, recently, another RCT on treating pneumonia patients with simvastatin did not support the statin administration for viral pneumonia [33].

Vandermeer et al. [32] evaluated the relationship between statin administration and mortality in hospitalized patients. For the first time, he used the data from CDC Emerging Infections Program (EIP) of influenza hospitalization surveillance that amassed information on hospitalized patients with laboratory-confirmed influenza. It was shown that statins decrease death among certain cases. Other published studies, evaluating the role of statins in reducing mortality from sepsis and community-acquired pneumonia [66, 67], suggest that their findings are conceivable.

Not all epidemiological studies are in favor of statin beneficiary effects on influenza-related illnesses. The study of Fleming et al. [27] did not find any benefit to statin use on the incidence of acute respiratory infections [27]. Their study included influenza-like illnesses such as acute bronchitis, pneumonia, and upper respiratory infections; however, they did not include earlier and severer conditions of influenza infections. In an observational study, Kwong et al. [26] evaluated the association between statins and pneumonia and influenza hospitalizations in elderly patients using a cohort study data from ten influenza seasons in Ontario, Canada. They identified trivial protective effects of statins averse to influenza mortality. They suggested that statins do not considerably reduce the morbidity and mortality of influenza. Some studies even showed the recurrence of the symptoms after discontinuing statin treatment [31, 34].

Several limitations may affect the results of this study. Firstly, in an observational study the effects of unmeasured confounders cannot be eliminated. Secondly, their outcomes were nonspecific and may be because of various causes other than influenza. Thirdly, they did not have any information on medications used while patients were being hospitalized. And, finally, they used health administrative data to assess covariates with dubious accuracy of diagnostic codes. Conversely, some observational studies have suggested a potential beneficiary role for statins in the treatment of sepsis and community-acquired pneumonia [66, 67]. A summary of the human studies was shown in Table 1.

It is necessary that people who have documented medical treatments in large administrative databases should undertake a comprehensive approach on the effects of statins in both retrospective cohort and case-control studies. Albeit, it is still ambiguous if these medications are truly beneficial [68]; the epidemiological findings can be used to generate hypotheses for a wide range of laboratory and clinical studies. Based on all current information, statins may be promising agents in preventing severe disease outcomes, such as death. However, these agents may not play a dominant role in reducing infections or minor illnesses. Thus, statin's prescription should be done cautiously, with regards to the background of the patient's disease. Moreover, to improve outcomes, statin dosage, duration of treatment, and also conjunction therapy with other antivirals should be respected as well [69].

Hence, scientists should thoroughly study statins to elucidate the potential of these agents as one of the alternative drugs in dominating outbreaks of influenza A virus infections in future.

## 6. Conclusion

Influenza-related illnesses are acute diseases that affect most of the human population, with various outcomes ranging from impaired quality of life to fatal infections. Despite contradictory results from various studies, statins are inexpensive and easily available medications in developing countries. They have the potential to be used as prophylactic medicine or therapy against influenza disease. Surely, they will contribute to the protection against inflammation-associated diseases, by controlling the immune system's cytokine overexpression and modulating the intense inflammatory response.

Therefore, more comprehensive epidemiological, functional, and molecular research on the interactions between statins and influenza virus and host immune responses needs to be carried out. This will lead to the development of more potent statins based on better profiles of bioavailability, which will help open new promising therapies for looming and imminent outbreaks of influenza. Lastly, comprehensive clinical studies should verify the benefits of statins for the treatment of influenza-related illnesses.

## Figures and Tables

**Table 1:** Human studies on the efficacy of using statins in acute respiratory patients.

Reference	Summary of the study	Conclusions
Frost et al., 2007 [25]	Investigating the relation of mortality risk and survival time with statin therapy in influenza and COPD patients.	Moderate dosage of statin treatment (≤4 mg/d) significantly reduced the mortality rate and survival time (*P* < 0.05).

Kwong et al., 2009 [26]	A cohort study of association between statins and pneumonia and influenza hospitalizations in elderly patients using data from ten influenza seasons in Ontario, Canada.	No considerable reduction in morbidity and mortality of influenza has been observed in statin-treated patients.

Fleming et al., 2010 [27]	Prospective study of the effects of statin on acute respiratory infections in primary care.	No beneficiary effects of statins on respiratory diseases have been observed. However, there was a kind of synergistic relation between statins and influenza vaccines.

Carrillo-Esper et al., 2011 [28]	Retrospective study of 26 patients with severe influenza in H1N1 influenza virus outbreak in 2009.	Combination treatments with methyl prednisolone, activated protein C, and statins had significantly increased the survival (*P* < 0.05).

Viasus et al., 2011 [29]	Cohort study of patients in pandemic influenza A (H1N1), 2009, complicated by pneumonia.	None of the therapies (corticosteroids, macrolides, and statins) were found effective to reduce the risk of severe disease.

Makris et al., 2011 [30]	A randomized controlled trial of ventilator associated pneumonia with oral pravastatin sodium.	This study evidenced that pravastatin may ameliorate the frequency of viral pneumonia.

Yende et al., 2011 [31]	Association of statin usage on clinical outcome and circulating biomarkers of community-acquired pneumonia patients.	Only a modest change in circulating biomarkers has been observed in statin-treated patients.

Vandermeer et al., 2012 [32]	Investigating the relation of statin therapy with influenza related death in laboratory confirmed patients of 59 countries in 10 states.	Statins may have beneficiary effects on reducing mortality in hospitalized influenza patients.

Papazian et al., 2013 [33]	A randomized controlled trial of ventilator associated pneumonia with simvastatin.	The result of this study does not support positive effects of simvastatin against ventilator associated pneumonia patients.

Kruger et al., 2013 [34]	A randomized controlled trial of the effects of atorvastatin on critically ill sepsis patients.	This cohort study showed that continuation of atorvastatin is associated with survival improvement.

## Abbreviations

CDC: Center for Disease Control and Prevention
CrFK: Crandell feline kidney
EIP: Emerging Infections Program
FPP: Farnesyl pyrophosphate
GGPP: Geranylgeranyl pyrophosphate
HMG-CoA: Hydroxyl methylglutaryl-coenzyme A
MDCK: Madin-Darby Canine Kidney
RCT: Randomized Controlled Trial.
